# Effect of Vagus Nerve Stimulation on Blood Inflammatory Markers in Children with Drug-Resistant Epilepsy: A Pilot Study

**DOI:** 10.3390/children9081133

**Published:** 2022-07-29

**Authors:** Valentina Baro, Maria Vittoria Bonavina, Francesco Saettini, Giovanna D’Amico, Andrea Trezza, Luca Denaro, Daniele Grioni, Andrea Landi

**Affiliations:** 1 Academic Neurosurgery, Department of Neuroscience, University of Padova, 35128 Padova, Italy; luca.denaro@unipd.it (L.D.); andrea.landi@unipd.it (A.L.); 2 Department of Women’s and Children’s Health, University Hospital of Padova, 35128 Padova, Italy; mariavittoriabonavina@gmail.com; 3 Department of Pediatric Onco-Hematology, San Gerardo Hospital, Fondazione MBBM, Università degli Studi di Milano-Bicocca, 20900 Monza, Italy; francescosaettini@yahoo.it; 4 Immunology and Cell Therapy, Centro Ricerca Tettamanti, Paediatric Clinic, University of Milan Bicocca/MBBM, 20900 Monza, Italy; giovanna.damico@asst-monza.it; 5 Neurosurgery Unit, San Gerardo Hospital, 20900 Monza, Italy; andrea.trezza@gmail.com; 6 Epilepsy Unit, Villa Santa Maria, Child Care Center-Neuropsychiatric Rehabilitation Center, 22038 Monza, Italy; d.grioni@hotmail.it

**Keywords:** vagus nerve stimulation, drug-resistant epilepsy, children, neuromodulation, neuroinflammation, cytokines

## Abstract

Background: Since one of the suggested mechanisms of action of VNS on epilepsy is the reduction of central inflammation, we carried out a comprehensive analysis of blood inflammatory markers in children considered for VNS surgery. Materials and methods: Five pediatric patients were studied. An extensive analysis of blood inflammatory markers was performed before surgery (T0) and six weeks after VNS implantation (T1). An epileptological outcome was obtained according to the McHugh score. Results: The variations of IgA, IgE, IgG, CD19, and PTX3 displayed a tendency toward a positive statistical correlation between T0 and T1. According to McHugh score, the patients were divided into Group 1 (i.e., Class I) and Group 2 (i.e., Classes II and III). IL-1β and PTX-3 tended to decrease more in Group 1, while TNF-α decreased in Group 2 (−56.65%) and slightly increased (+3.61%) in Group 1 at T1 without statistical correlation. Conclusions: The variation of IL-1β and PTX-3 seem to be related to a better outcome; thus, they do not reach statistical significance. A larger series of patients is needed to determine whether biochemical changes could relay with the clinical improvement of epilepsy.

## 1. Introduction

Epilepsy is one of the most common chronic neurologic disorders, affecting approximately 50 million people worldwide, with an average annual incidence of 67.77 cases/100,000 inhabitants [1]. Despite adequate treatment with antiseizure drugs, about one-third of the patients, mostly children, prove to be resistant to current medications and are therefore considered to have a drug-resistant epilepsy (DRE). When resective surgery was not feasible, vagus nerve stimulation (VNS) was proposed as an alternative treatment for this cohort of patients, showing a significant reduction in seizure frequency and duration [2,3,4,5,6,7,8,9,10,11,12,13]. Over the last decade, an increasing number of experimental and clinical evidence has suggested the hypothesis of an inflammatory genesis for epilepsy. In light of this large body of evidence, the inflammation acquired a causative role with a well-recognized inflammatory cytokine profile in epileptogenesis (e.g., IL-1β, IL-6, TNF, lipopolysaccharide, and prostaglandin E2) [14,15,16,17,18,19,20,21,22,23,24]. In fact, the potential role of inflammation in epilepsy was highlighted in the patients affected by Rasmussen encephalitis [25], and the immune system activation is known to be fundamental in patients with febrile seizures [26]. Moreover, in patients with drug-resistant temporal lobe epilepsy and cortical dysplasia who underwent epilepsy surgery, several inflammatory mediators have been detected in the resected brain tissue [14,16]. Recent research suggested an important anti-inflammatory action of the central nervous system, for which the term “inflammatory reflex” was coined [27], having both immunosensing and immunosuppressing functions that are accomplished through three pathways: the hypothalamic–pituitary–adrenal axis, the cholinergic anti-inflammatory pathway (CAP), and the splenic sympathetic anti-inflammatory pathway. Furthermore, the involvement of the vagus nerve (VN) in these pathways has been described in vivo both in systemic and localized inflammation [27,28,29,30,31,32,33]. According to these complex findings, some authors have applied low-frequency (10 Hz) VNS for the treatment of chronic inflammatory diseases such as Crohn’s disease and rheumatoid arthritis (RA), both in animal model and in human patients, with promising results [32,33,34,35,36]. Bonaz et al. treated with low-frequency VNS seven patients affected by Crohn’s disease, obtaining a clinical, biological, and endoscopic improvement in five out of seven patients after 6 months of follow-up [37]. Similarly, Koopman et al. investigated the effects of VNS on the plasmatic levels of cytokines in patients affected by RA showing an inhibition of peripheral blood production of TNF-α, IL-1β, and IL-6, correlated with an improvement of the severity of the disease [33]. Furthermore, these authors also studied seven adult patients with epilepsy treated with VNS in whom a reduction of IL-6, IL-1β, and TNF blood production was observed. Since DRE affects mostly children, who benefit from VNS, in this paper, we investigated the inflammation blood markers in a cohort of pediatric patients with the abovementioned characteristics. Our aims were (a) to evaluate whether VNS causes a reduction of inflammatory markers in children, as observed in adults; (b) to investigate whether the biochemical changes would be related to epilepsy outcome; and (c) to identify predictive blood markers of the efficacy of VNS.

## 2. Materials and Methods

We prospectively studied five consecutive pediatric patients who underwent VNS placement due to DRE at the Neurosurgery Unit of the San Gerardo Hospital, Fondazione MBBM, Università degli Studi di Milano-Bicocca from November 2016 to November 2017. In all cases, the preoperative evaluation followed the ILAE international guidelines for resective epilepsy surgery in children [38]. The M:F ratio was 4:1, and the mean age at surgery 10.4 ± 4.7 years. Preoperative data are shown in Table 1. All patients had no history of inflammatory or autoimmune disorders.

Peripheral blood was collected at admission (T0); testing total leukocytes; lymphocytes; CD3, CD4, CD8, CD19, CD56, and CD4/CD8 T cells; platelets; erythrocyte sedimentation rate; C-reactive protein (CRP); immunoglobulins (IgG, IgA, IgM, and IgE); cytokines (TNF-α, IL-1β); and pentraxin-3 (PTX-3). IL-6 was not assessed for technical issues. Lipopolysaccharide (LPS) was added to the whole blood to stimulate the production of TNF by monocytes for 4 h, and blood samples were incubated at 37 °C in a steamy environment, at 5% CO_2_, for 24 h, with and without LPS stimulation (100 ng/mL) [39]. Cytokines levels were detected and quantitatively determined by enzyme-linked immunosorbent assay (ELISA), (R & D Systems, Minneapolis, MN, USA).

Surgery was performed according to the standard surgical technique, and the implanted devices were M103 and M106 (LivaNova Co., Plano, TX, USA) in 4 patients and 1 patient, respectively. The VN was stimulated during surgery to measure electrode impedance and to verify device function. The stimulation was then turned off and activated 25.6 ± 7.1 days after surgery, with standard parameters (electric current pulses of 250 μs duration at 10 Hz and an output current of 1.0 mA; duty cycle of 30 s ON and 3 min OFF), further modulated according to the clinical response at medical consultations.

According to Koopman et al. [40], a second blood test, analyzing the same preoperative panel of inflammatory mediators, was performed 42 days after surgery (T1). The epileptological outcome at last follow-up was defined by applying the dedicated modified McHugh score (mMH) [41] (Table 2), and the patients were divided into two groups depending on the clinical response: mMH Class I for Group 1 and mMH Class > I for Group 2.

This dichotomization was applied (a) to investigate the correlation between pre/post-stimulation inflammatory changes and the epilepsy outcome and (b) to identify the presence of predictive inflammatory markers of the efficacy of VNS.

### Statistical Analysis

All statistical data were analyzed by using TIBCO Statistica^®^ 13.3.0. The results are presented as mean ± SD, or as number of cases. Correlation between results in T0 and results in T1 was performed by using the Wilcoxon test. Furthermore, the Mann–Whitney U test was applied to compare the results obtained in Group 1 and Group 2. Statistical significance was set at *p* < 0.05.

## 3. Results

### 3.1. Clinical Findings

The epileptological outcome was defined at last follow-up; the mean length was 65.6 ± 7.6 months. Two patients achieved mMH Class I, thus identifying Group 1; one patient improved up to mMH Class II, while the remaining showed a limited improvement classified mMH Class III, thus identifying Group 2 of the cohort of patients. At the last follow-up, the EEG was normalized for both patients in Group 1, while the EEG findings improved for the patient in mMH Class II. The antiseizure therapy was reduced in all but one patient. All postoperative clinical findings concerning EEG, mMH class, and AEDs modifications are reported in Table 3. The final VN stimulation parameters were achieved 2.7 ± 0.8 months after its activation, and they are summarized in Table 4.

### 3.2. Biochemical Findings

The analysis of inflammatory parameters of all the patients comparing T0 and T1 did not show any statistical correlations. The variations of IgA, IgE, IgG, CD19, and PTX3 displayed a tendency toward a positive statistical correlation, although without reaching significance. Values are presented in Table 5. According to the mMH score, we divided the patients in two groups, as above mentioned. The inflammatory parameters did not show a statistical difference between Group 1 and Group 2. Focusing on IL-1β, TNF-α, and PTX3 in order to be comparable with the previous literature, we observed that IL-1β and PTX3 decreased more in Group 1 than in Group 2 at T1 (−24.8% vs. −2.25% and −47.38 vs. −11.01% respectively), while TNF-α decreased in Group 2 (−56.65%) and slightly increased in the other (+3.61%), without reaching statistical significance (Table 6).

## 4. Discussion

Vagal nerve stimulation is widely used to treat DRE, and it is generally well tolerated and effective both in adults and in children [2,3,4,5,6,7,8,9,10,11,12,13]. This effect is achieved by activating, with high-frequency electric stimulation (20–30 Hz) [42], the vagal afferents via the polysynaptic pathway from the nucleus tractus solitarius to cortical regions, involving components of the central autonomic system, the limbic system, and the locus coeruleus [43,44,45].

The role of VN in the modulation of the visceral activity has been widely described both in animal models and in humans affected by inflammatory digestive disorders, as well as in extra-digestive inflammatory diseases in which TNF-α is a key factor for their pathobiology [27,28,29,30,31,33,34,35,36,46]. In this scenario, VN plays a pivotal role with its efferent, modulating the CAP, which has an anti-TNF-α action, inhibiting its production by monocyte and macrophage. This effect is obtained with a low frequency of stimulation (1–10 Hz) [28,47,48].

Koopman et al. studied seven adult patients with epilepsy implanted with VNS, applying stimulation parameters suited to activate the inflammatory reflex (single 30 s stimulation at 1.0 mA output current, 20 Hz pulse frequency, and 500 µs pulse duration) and then observing an inhibition of TNF-α, IL-6, and IL-1β that occurred early after switching on the device [33]. Unfortunately, the study did not evaluate the clinical effect of these parameters of stimulation on the epilepsy.

We designed this study to investigate inflammatory changes in pediatric patients with DRE undergoing VNS with standard stimulation protocol for epilepsy [42]. Considering our objectives, we found several differences in the modulation of inflammatory markers comparing the previous study involving adults [33,40,49]. In our cohort of patients, we found higher values at T1 of immunoglobulins, with a tendency toward a positive statistical correlation. Since antibodies’ production is dependent on vagal innervation, this could suggest that the adaptive immune system is under cholinergic control [50]. When analyzing the patients after the dichotomization based on the clinical response, we observed that TNF-α decreased in patients with a partial effect of the stimulation, while in the others, we found even a slight increase, a finding which is counterintuitive and also contrasts with previous reports. Nevertheless, in patients with remission of seizure, we identified a greater reduction of IL-1β and PTX3, but the lack of statistical significance does not allow us to draw any conclusion from these data.

Forasmuch as the anti-epileptic and anti-inflammatory modulating function of VN are activated by different electrical stimulation parameters, the effect on cytokines’ modulation during high-frequency VNS could have been partial and/or not effective. Therefore, this work did not take into consideration that we could have overstimulated the CAP, with unknown clinical implications. Furthermore, the duration of the stimulation phase for epilepsy differs from the one used to obtain the stimulation of the anti-inflammatory reflex in preclinical and clinical study [33], representing another variable to be considered. The limited sample size constitutes another limitation of this study, undermining the statistical power of the analysis. Further investigations on anti-inflammatory parameters in patients affected by DRE treated with VNS are needed to better understand the modulation of CAP during high-frequency stimulation and possible blood inflammatory markers’ variations.

## 5. Conclusions

This preliminary study tried to evaluate the cholinergic anti-inflammatory reflex mediated by vagal nerve stimulation in pediatric patients with drug-resistant epilepsy. IL-1β and PTX-3 seem to be related to a better outcome, and are, thus, without statistical significance. The parameters of stimulation chosen were those of standard protocol for epilepsy rather those used to obtain the effects on peripheral blood inflammatory elements, highlighting the different modulation of the vagal nerve fibers according to the frequency of stimulation. A larger series of patients is needed to determine whether biochemical changes could relay with the clinical improvement of epilepsy.

## Figures and Tables

**Table 1:** Preoperative characteristics of the patients.

Patient	Age at Surgery	Sex	Type of Epilepsy	MRI Findings	Preoperative EEG
1	2.5 years	M	West syndrome	No pathological findings	Hypsarrythmia
2	13.8 years	M	Focal seizures	Post ischemic area in the left temporal, parietal, occipital lobes	Multifocal
3	9.2 years	M	Focal	Post ischemic area in the right frontal, temporal, parietal lobes	Multifocal
4	15.2 years	F	Generalized	No pathological findings.	Generalized
5	12 years	M	Focal and generalized	Post ischemic area in the right frontal, temporal, insular, parietal lobes	Multifocal

**Table 2:** Modified McHugh classification.

Class	Reduction in Seizure Frequency
Class I	80–100%
Class II	50–79%
Class III	<50%
Class IV	Magnet benefit only
Class V	No improvement

**Table 3:** McHugh score, EEG findings at last follow-up and anti-epileptic drug therapy modifications.

Patient	McHugh Score	EEG Findings	Preoperative Antiseizure Drugs	Antiseizure Drugs at Last Follow-Up	Last Follow-Up (months)
1	I	Normal	Steroids; many antiepileptic drugs were used (including Vigabatrin) without benefit	Vigabatrin	54.7
2	II	Improved	LTG, VPA, TPM	LTG, VPA	59.7
3	III	Unchanged	CBZ	OXC	65.7
4	I	Normal	LTG, ETS	LTG	73
5	III	Unchanged	VPA, OXC	VPA-OXC-PHT-BDZ	74.7

BDZ: Benzodiazepines; CBZ: Carbamazepine; ETS: etoposide; LTG: lamotrigine; OXC: oxcarbazepine; PTH: phenitoine; TPM: topiramate; VPA: valproate.

**Table 4:** Stimulation parameters at last follow-up.

Patient	Output Current	Frequency	ON/OFF Cycle	Pulse Width
1	1.5 mA	30 Hz	30 sec ON-1.8 min OFF	500 μs
2	1.75 mA	30 Hz	30 sec ON-3 min OFF	500 μs
3	2.25 mA	30 Hz	30 sec ON-1.8 min OFF	500 μs
4	1.75 mA	30 Hz	30 sec ON-3 min OFF	250 μs
5	2 mA	30 Hz	30 sec ON-3 min OFF	500 μs

**Table 5:** Changes in immunology and inflammation parameters across time points. Data are shown as mean ± standard deviation.

	Day 0	Day 42	
**CRP**	0.48 ± 0.76	0.49 ± 0.53	**ns**
**ESR**	24.60 ± 20.33	14.40 ± 13.59	**ns**
**WBC**	8020 ± 3451	8455 ± 2539	**ns**
**Lymphocytes**	3134 ± 1523	4270 ± 2527	**ns**
**CD3+**	2211 ± 1560	2598 ± 1346	**ns**
**CD4+/CD8+ ratio**	2.04 ± 0.49	2.32 ± 0.80	**ns**
**CD4+**	1114 ± 522	1647 ± 926	**ns**
**CD8+**	538 ± 199	725 ± 360	**ns**
**CD19+**	773.3 ± 626.3	988.7 ± 978.9	***p* = 0.067**
**CD56+**	303 ± 193	533 ± 234	**ns**
**IgG**	917.2 ± 90.5	1043 ± 146.1	***p* = 0.067**
**IgA**	176.3 ± 66.1	194.0 ± 65.0	***p* = 0.067**
**IgM**	160 ± 24	132 ± 43	**ns**
**IgE**	9.867 ± 10.1535	13.933 ± 11.0024	***p* = 0.11**
**TNF-α**	67.9 ± 64.18	42.84 ± 31.36	**ns**
**IL-1β**	2.21 ± 0.58	1.955 ± 0	**ns**
**PTX3**	1005.9 ± 939.1	652.8 ± 477.7	***p* = 0.14**

CRP: c-reactive protein. ESR: erythrocyte sedimentation rate; WBC: white blood cells; CD: cluster of differentiation; Ig: immunoglobulin.

**Table 6:** Comparison between IL-1β, TNF-α and PTX3 between Group 1 and Group 2. Data are shown as mean ± standard deviation.

Variable	Group 1 (2 pts)	Group 2 (3 pts)	
IL-1β T0	2.60 ± 0.91	2.0 ± 0	
IL-1β T1	1.955 ± 0	1.955 ± 0	**ns**
TNF-α T0	55.62 ± 34.33	76.08 ± 86.01	
TNF- α T1	57.63 ± 55.27	32.98 ± 8.65	**ns**
PTX 3 T0	3591.9 ± 1730.99	1757.31 ± 621.18	
PTX 3 T1	1889.55 ± 151.25	1563.81 ± 332.84	**ns**

Abbreviations: pts: patients; IL-1β: interleukin 1β; TNF-α: tumor necrosis factor-α; PTX3: pentraxin 3.

## Data Availability

All data are available in the text.

## Abbreviations

CAP, cholinergic anti-inflammatory pathway; CRP, C-reactive protein; DRE, drug-resistant epilepsy; mMH, modified McHugh score; ILAE, international league against epilepsy; IL, interleukin; LPS, lipopolysaccharide; PTX-3, pentraxin-3; RA, rheumatoid arthritis TNF, tumor necrosis factor; VN, vagus nerve; VNS, vagus nerve stimulation.

## References

[1] Fiest K.M., Sauro K.M., Wiebe S., Patten S.B., Kwon C.S., Dykeman J., Pringsheim T., Lorenzetti D.L., Jetté N. (2017). Prevalence and incidence of epilepsy: A systematic review and meta-analysis of international studies. Neurology.

[2] Alexopoulos A.V., Kotagal P., Loddenkemper T., Hammel J., Bingaman W.E. (2006). Long-term results with vagus nerve stimulation in children with pharmacoresistant epilepsy. Seizure.

[3] Healy S., Lang J., Naude J.T.W., Gibbon F., Leach P. (2013). Vagal nerve stimulation in children under 12 years old with medically intractable epilepsy. Child’s Nerv. Syst..

[4] Berg A.T., Rychlik K., Levy S.R., Testa F.M. (2014). Complete remission of childhood-onset epilepsy: Stability and prediction over two decades. Brain.

[5] Orosz I., McCormick D., Zamponi N., Varadkar S., Feucht M., Parain D., Griens R., Vallée L., Boon P., Rittey C. (2014). Vagus nerve stimulation for drug-resistant epilepsy: A European long-term study up to 24 months in 347 children. Epilepsia.

[6] Terra V.C., Furlanetti L.L., Nunes A.A., Thomé U., Nisyiama M.A., Sakamoto A.C., Machado H.R. (2014). Vagus nerve stimulation in pediatric patients: Is it really worthwhile?. Epilepsy Behav..

[7] Grioni D., Landi A., Gasperini S., Trezza A., Fiori L., Rigoldi M., Parini R., Sganzerla E.P. (2015). Vagal Nerve Stimulation in the Treatment of Drug-Resistant Epileptic Encephalopathies in Inborn Errors of Metabolism. Child Neurol. Open.

[8] Grioni D., Landi A., Fiori L., Sganzerla E.P. (2018). Does emergent implantation of a vagal nerve stimulator stop refractory status epilepticus in children?. Seizure.

[9] Soleman J., Stein M., Knorr C., Datta A.N., Constantini S., Fried I., Guzman R., Kramer U. (2018). Improved quality of life and cognition after early vagal nerve stimulator implantation in children. Epilepsy Behav..

[10] Boon P., De Cock E., Mertens A., Trinka E. (2018). Neurostimulation for drug-resistant epilepsy: A systematic review of clinical evidence for efficacy, safety, contraindications and predictors for response. Curr. Opin. Neurol..

[11] Grioni D., Landi A. (2019). Does Vagal Nerve Stimulation Treat Drug-Resistant Epilepsy in Patients with Tuberous Sclerosis Complex?. World Neurosurg..

[12] Starnes K., Miller K., Wong-Kisiel L., Lundstrom B.N. (2019). A Review of Neurostimulation for Epilepsy in Pediatrics. Brain Sci..

[13] Toffa D.H., Touma L., El Meskine T., Bouthillier A., Nguyen D.K. (2020). Learnings from 30 years of reported efficacy and safety of vagus nerve stimulation (VNS) for epilepsy treatment: A critical review. Seizure.

[14] Vezzani A., Granata T. (2005). Critical Review Brain Inflammation in Epilepsy: Experimental and Clinical Evidence. Epilepsia.

[15] Ravizza T., Gagliardi B., Noé F., Boer K., Aronica E., Vezzani A. (2008). Innate and adaptive immunity during epileptogenesis and spontaneous seizures: Evidence from experimental models and human temporal lobe epilepsy. Neurobiol. Dis..

[16] Choi J., Nordli D.R., Alden T.D., DiPatri A., Laux L., Kelley K., Rosenow J., Schuele S.U., Rajaram V., Koh S. (2009). Cellular injury and neuroinflammation in children with chronic intractable epilepsy. J. Neuroinflamm..

[17] Riazi K., Galic M.A., Pittman Q.J. (2010). Contributions of peripheral inflammation to seizure susceptibility: Cytokines and brain excitability. Epilepsy Res..

[18] Aronica E., Ravizza T., Zurolo E., Vezzani A. (2012). Astrocyte immune responses in epilepsy. Glia.

[19] Librizzi L., Noè F., Vezzani A., de Curtis M., Ravizza T. (2012). Seizure-induced brain-borne inflammation sustains seizure recurrence and blood-brain barrier damage. Ann. Neurol..

[20] Devinsky O., Vezzani A., Najjar S., De Lanerolle N.C., Rogawski M.A. (2013). Glia and epilepsy: Excitability and inflammation. Trends Neurosci..

[21] Vezzani A., Aronica E., Mazarati A., Pittman Q. (2011). Epilepsy and brain inflammation. Exp. Neurol..

[22] Vezzani A., Friedman A., Dingledine R.J. (2012). The role of inflammation in epileptogenesis. Neuropharmacology.

[23] de Vries E.E., Munckhof B.V.D., Braun K.P., van Royen-Kerkhof A., de Jager W., Jansen F.E. (2016). Inflammatory mediators in human epilepsy: A systematic review and meta-analysis. Neurosci. Biobehav. Rev..

[24] Vezzani A., Lang B., Aronica E. (2015). Immunity and Inflammation in Epilepsy. Cold Spring Harb. Perspect. Med..

[25] Rasmussen T., Olszewski J., Lloyd-Smith D. (1958). Focal seizures due to chronic localized encephalitis. Neurology.

[26] Dubé C.M., Brewster A.L., Richichi C., Zha Q., Baram T.Z. (2007). Fever, febrile seizures and epilepsy. Trends Neurosci..

[27] Tracey K.J. (2002). The inflammatory reflex. Nature.

[28] Borovikova L.V., Ivanova S., Zhang M., Yang H., Botchkina G.I., Watkins L.R., Wang H., Abumrad N., Eaton J.W., Tracey K.J. (2000). Vagus nerve stimulation attenuates the systemic inflammatory response to endotoxin. Nature.

[29] Tracey K.J. (2007). Physiology and immunology of the cholinergic antiinflammatory pathway. J. Clin. Investig..

[30] Johnston G.R., Webster N.R. (2009). Cytokines and the immunomodulatory function of the vagus nerve. Br. J. Anaesth..

[31] Martelli D., McKinley M., McAllen R. (2014). The cholinergic anti-inflammatory pathway: A critical review. Auton. Neurosci..

[32] Bonaz B., Sinniger V., Pellissier S. (2016). Anti-inflammatory properties of the vagus nerve: Potential therapeutic implications of vagus nerve stimulation. J. Physiol..

[33] Koopman F.A., Chavan S.S., Miljko S., Grazio S., Sokolovic S., Schuurman P.R., Mehta A.D., Levine Y.A., Faltys M., Zitnik R. (2016). Vagus nerve stimulation inhibits cytokine production and attenuates disease severity in rheumatoid arthritis. Proc. Natl. Acad. Sci. USA.

[34] Meregnani J., Clarençon D., Vivier M., Peinnequin A., Mouret C., Sinniger V., Picq C., Job A., Canini F., Jacquier-Sarlin M. (2011). Anti-inflammatory effect of vagus nerve stimulation in a rat model of inflammatory bowel disease. Auton. Neurosci..

[35] Hoover D.B. (2017). Cholinergic modulation of the immune system presents new approaches for treating inflammation. Pharmacol. Ther..

[36] Jin H., Guo J., Liu J., Lyu B., Foreman R.D., Yin J., Shi Z., Chen J.D. (2017). Anti-inflammatory effects and mechanisms of vagal nerve stimulation combined with electroacupuncture in a rodent model of TNBS-induced colitis. Am. J. Physiol. Gastrointest. Liver Physiol..

[37] Bonaz B., Sinniger V., Pellissier S. (2017). The Vagus Nerve in the Neuro-Immune Axis: Implications in the Pathology of the Gastrointestinal Tract. Front. Immunol..

[38] Jayakar P., Gaillard W.D., Tripathi M., Libenson M.H., Mathern G.W., Cross J.H. (2014). Diagnostic test utilization in evaluation for resective epilepsy surgery in children. Epilepsia.

[39] Rosas-Ballina M., Goldstein R.S., Gallowitsch-Puerta M., Yang L., Valdés-Ferrer S.I., Patel N.B., Chavan S., Al-Abed Y., Yang H., Tracey K.J. (2009). The Selective α7 Agonist GTS-21 Attenuates Cytokine Production in Human Whole Blood and Human Monocytes Activated by Ligands for TLR2, TLR3, TLR4, TLR9, and RAGE. Mol. Med..

[40] Koopman F.A., Van Maanen M.A., Vervoordeldonk M.J., Tak P.P. (2017). Balancing the autonomic nervous system to reduce inflammation in rheumatoid arthritis. J. Intern. Med..

[41] McHugh J.C., Singh H.W., Phillips J., Murphy K., Doherty C.P., Delanty N. (2007). Outcome Measurement after Vagal Nerve Stimulation Therapy: Proposal of a New Classification. Epilepsia.

[42] Johnson R.L., Wilson C.G. (2018). A review of vagus nerve stimulation as a therapeutic intervention. J. Inflamm. Res..

[43] Beekwilder J.P.B.T. (2010). Overview of the clinical application of vagus nerve stimulation. J. Clin. Neurophysiol..

[44] Groves D.A., Brown V.J. (2005). Vagal nerve stimulation: A review of its applications and potential mechanisms that mediate its clinical effects. Neurosci. Biobehav. Rev..

[45] Krahl S.E., Clark K.B., Smith D.C., Browning R.A. (1998). Q International League Against Epilepsy Locus Coeruleus Lesions Suppress the Seizure-Attenuating Effects of Vagus Nerve Stimulation. Epilepsia.

[46] Bonaz B., Bazin T., Pellissier S. (2018). The Vagus Nerve at the Interface of the Microbiota-Gut-Brain Axis. Front. Neurosci..

[47] De Jonge W.J., Van Der Zanden E.P., The F.O., Bijlsma M.F., Van Westerloo D.J., Bennink R.J., Berthoud H.-R., Uematsu S., Akira S., van den Wijngaard R.M. (2005). Stimulation of the vagus nerve attenuates macrophage activation by activating the Jak2-STAT3 signaling pathway. Nat. Immunol..

[48] Bernik T.R., Friedman S.G., Ochani M., DiRaimo R., Ulloa L., Yang H., Sudan S., Czura C.J., Ivanova S.M., Tracey K.J. (2002). Pharmacological Stimulation of the Cholinergic Antiinflammatory Pathway. J Exp. Med..

[49] Bonaz B., Sinniger V., Hoffmann D., Clarençon D., Mathieu N., Dantzer C., Vercueil L., Picq C., Trocmé C., Faure P. (2016). Chronic vagus nerve stimulation in Crohn’s disease: A 6-month follow-up pilot study. Neurogastroenterol. Motil..

[50] Buijs R.M., Van Der Vliet J., Garidou M.-L., Huitinga I., Escobar C. (2008). Spleen Vagal Denervation Inhibits the Production of Antibodies to Circulating Antigens. PLoS ONE.

